# Complementary and alternative medicine use by pediatric oncology patients before, during, and after treatment

**DOI:** 10.1186/s12906-021-03271-9

**Published:** 2021-03-18

**Authors:** Emmanuelle Lüthi, Manuel Diezi, Nadia Danon, Julie Dubois, Jérôme Pasquier, Bernard Burnand, Pierre-Yves Rodondi

**Affiliations:** 1 Institute of Family Medicine, University of Fribourg, Route des Arsenaux 41, 1700 Fribourg, Switzerland; 2 Center for Primary Care and Public Health (Unisanté), University of Lausanne, Lausanne, Switzerland; 3 Pediatric Onco-Hematology Unit, Lausanne University Hospital, Lausanne, Switzerland; 4 Department of Anesthesiology, Lausanne University Hospital, Pain Center and Center for Integrative and Complementary Medicine, Lausanne, Switzerland

**Keywords:** Pediatric oncology, Complementary and alternative medicine, Childhood cancer, Physician patient communication, Survivorship

## Abstract

**Background:**

The prevalence of complementary and alternative medicine (CAM) use and the modalities used by pediatric oncology patients vary widely across studies. In addition, the changes in the use of CAM over the course of treatment are understudied. Thus, this study aimed to explore (1) CAM use by pediatric oncology patients in relation to specific time intervals and (2) communication about CAM use between parents and oncologists.

**Methods:**

This retrospective cross-sectional study was conducted among parents of children diagnosed with cancer at a Swiss pediatric hematology-oncology center by means of an online questionnaire. Questions were related to their child’s CAM use over different time intervals, sources of information about CAM use, and communication with the oncologists.

**Results:**

Among 140 respondents, CAM was used by 54.3% of patients before diagnosis and 69.3% of patients after diagnosis. During each defined time interval, between 50 and 58.8% of the patients used at least one CAM. Homeopathy was the most popular CAM modality used during oncology treatment, during the first year after treatment, and between 1 and 5 years after the end of treatment. Osteopathy was the most popular CAM ≥5 years after the end of oncology treatment. Forty percent of respondents did not discuss CAM with their oncologist.

**Conclusions:**

The high prevalence of CAM use and the different trends of use during the oncology care pathway and afterward underline the need to increase communication about CAM in the pediatric oncology setting, notably regarding benefits and risks of interaction with oncology treatment.

**Supplementary Information:**

The online version contains supplementary material available at 10.1186/s12906-021-03271-9.

## Background

The prevalence of complementary and alternative medicine (CAM) use by pediatric oncology patients varies widely, ranging from 6 to 91% worldwide [1], with 53% use in Switzerland [2]. In 2013, it was estimated that about every second child in Europe had used CAM [3]. CAM is defined by the World Health Organization as “a broad set of health care practices that are not part of that country’s own tradition and are not integrated into the dominant health care system” [4]. However, as several definitions of CAM exist [5, 6], the CAM modalities and classifications proposed in questionnaires have varied between studies [1]. Time taken into consideration since diagnosis is also heterogeneous [7–13]. Indeed, the time considered could be a specific time [8, 9] or time intervals [13], related to disease evolution [10] and treatment [12], or an approximation such as “since diagnosis” or “after diagnosis” [2, 7, 11].

Interaction risks between pediatric oncology treatment and CAM have been highlighted in several studies [14–17]. Nonetheless, one study found that less than half of pediatric oncologists routinely asked their patients about CAM use, although 99% considered that being aware of the kind of CAM their patients used was important [18]. Disclosure of CAM use by the patient’s parents to the oncologist is also not standard, reportedly ranging from 34 to 78% [2, 7, 8, 10]. Parents of oncology patients most frequently consult family or friends [2, 7, 9, 11] for information about CAM.

On the one side, very few studies investigate CAM use according to different time intervals and data about CAM use among pediatric oncology patients in Switzerland are scarce. On the other side, available literature underlines the importance of communication between the patient’s parents and oncologist about CAM use in order to open up discussion concerning interaction risks. Thus, this study aimed to explore (1) CAM use by pediatric oncology patients in relation to specific time intervals and (2) communication about CAM use between parents and oncologists.

## Methods

### Study design

We conducted a retrospective quantitative cross-sectional survey that included pediatric oncology patients from a single academic medical center, the one of the two that treat those patients in the French-speaking part of Switzerland. Parents of patients were asked to fill in an online questionnaire about their opinions and experiences concerning their child’s CAM use.

### Setting and respondents

All patients (0–18 years old at the time of cancer diagnosis) who were being or had been treated at Lausanne University Hospital, Switzerland, between 2007 and 2017 were included (*n* = 477). In June 2018, their parents received an invitation by postal mail containing a study information sheet with a personal access code to the online questionnaire, and an informed consent form to be signed and sent back by post. As a reminder, a letter was sent 1 month later and a phone call 4 months later to non-responders. If the patients were deceased or in palliative care, their parents were not contacted for ethical reasons, as causing them to remember a deceased child is a painful exercise that had no place in this study. Parents of patients who did not understand French were excluded. Because this academic hospital is the national retinoblastoma center and receives around 50 patients from abroad every year, patients living outside Switzerland were excluded from the study, so as not to influence the results concerning the resident population.

Although we stated that the questionnaire had to be filled in by the parents with the help of their child, we eventually included questionnaires filled in only by the patients (*n* = 7) because all of them were ≥ 18 years old at the time of the study. The questionnaire was available online from June to October 2018.

### Variables

In the absence of a validated questionnaire, we developed one following a literature search [1, 2, 7, 11, 13, 19, 20] and in collaboration with epidemiologists and pediatric oncologists. A total of 23 questions were divided into four sections: (1) the child’s lifetime CAM use and use since diagnosis, (2) modalities of CAM used within specific time intervals, (3) sources of information used by the parents about CAM, and (4) communication about CAM between the patient’s parents and the pediatric oncologist (see Additional files 1, 2). Twenty-two types of CAM were listed as possible answers and the respondents could freely add up types of CAM.

We selected CAMs on the basis of previous Swiss studies on CAM use [21, 22] or conducted in pediatric oncology to compare results [2, 7, 8, 23]. In Switzerland, many CAMs are covered by private supplemental health insurance that include various conditions for reimbursement. We distinguished dietary supplements (vitamins and trace elements) from herbal medicine. In the second section, we defined five time intervals for CAM use: (1) before diagnosis, (2) during oncology treatment, (3) during the first year after the end of oncology treatment, (4) between 1 and 5 years after the end of oncology treatment, and (5) ≥5 years after the end of oncology treatment. We decided to define such time intervals because they may reflect distinct experiences of the disease. Moreover, the interaction risks differ if CAM is used during or after oncology treatment, and treatment side effects may also appear in the short or long term [24, 25].

The questionnaire was cognitively tested [26] by five parents with healthy children and five parents of pediatric oncology patients. The wording of the questions was only slightly modified after this step.

According to the quality-assessment tool developed by Bishop et al. [1] to improve the quality and comparability of questionnaires concerning CAM use in pediatric oncology, our questionnaire scored 12.5 of 18 points (69%). Only 4 of 29 studies had a higher percentage [1].

After consent was given, the patient’s personal data were included in our analysis (age, age at diagnosis, gender, date of diagnosis, type of cancer, oncologic treatment received, status of recurrence).

### Data sources

#### Comparison groups

We categorized respondents into two groups according to CAM use after cancer diagnosis exclusively: “CAM users” and “CAM non-users.” In the question, “*For each CAM used, please indicate when your child used it*,” any respondent who checked at least one box among the four time intervals after cancer diagnosis was considered a CAM user. The number of respondents included in the CAM users group had to be modified after data collection (32 respondents initially identified themselves as CAM non-users, but later on indicated CAM use during specific time intervals). Consequently, 32 respondents did not access three questions addressing communication about CAM use. After this correction, the final number of CAM users was 97 rather than 65.

### Risk of bias

Anonymized medical data of respondents and non-respondents (age, age at diagnosis, gender, type of cancer, and treatment received) were compared to estimate the risk of selection bias. There were no significant differences between patients in the respondents’ group and the non-respondents’ group (gender, type of cancer, treatment received), except for age at the time of the study and at diagnosis.

### Statistical analysis

Standard descriptive analyses (e.g., frequencies and percentages for categorical variables and means and standard deviations for continuous variables) were used to summarize socio-demographic variables, patient characteristics, and responses to the questionnaires. In all of these areas, we conducted comparisons between CAM users and non-users by using Fisher’s exact test and the Student’s t-test for categorical and continuous variables, respectively. For each CAM considered, the prevalence of use before diagnosis, during treatment, and during the first year after treatment were compared two by two for the group of CAM user. For this analysis, only patients who were at least during the first year after treatment were considered (in order to obtain paired data). Thereby, the prevalence was compared by using the McNemar test. Statistical significance was established at *p* < 0.05.

## Results

### Study sample

**Fig. 1:**
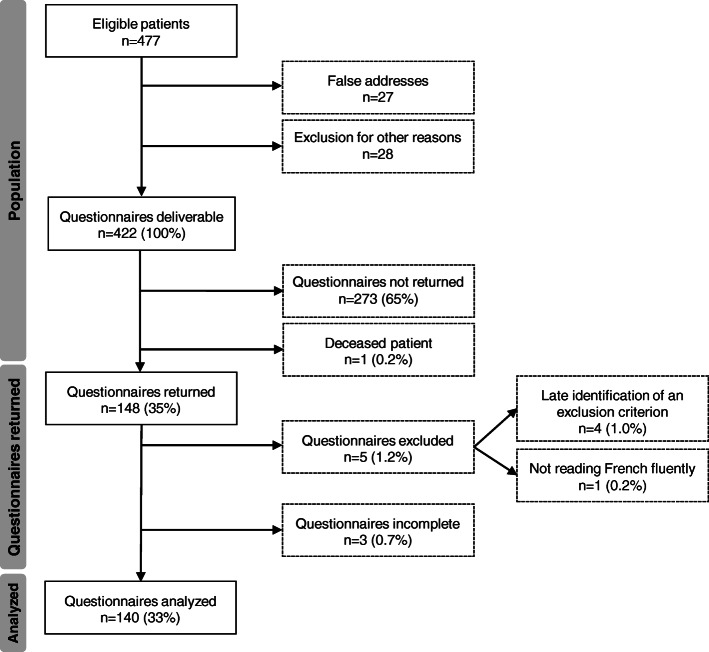
Flow diagram of the study

Of 421 eligible questionnaires, 148 were returned, of which 140 could be analyzed. The response rate was therefore 33%. Details are given in Fig. 1.

### Socio-demographic characteristics and medical data of patients

The socio-demographic characteristics and medical data of the patients are described in Table 1. The mean age (SD) of the patients at the time of the survey was 12.3 (5.4) years and the mean age at diagnosis was 7.1 (5.0) years. The gender proportion was equal. Fifteen patients were still under oncology treatment at the time of the study. Leukemia and malignant solid tumors were the most frequent diagnoses among patients and chemotherapy the most frequent treatment. Among CAM users, 80.4% had subscribed to CAM supplemental healthcare insurance, as well as half of the CAM non-users (*p* < 0.01).

**Table 1:** Socio-demographic characteristics and medical data of patients

	Total (***n*** = 140)	CAM users (***n*** = 97)	CAM non-users (***n*** = 43)	***p***-value^**c**^
Age, years, mean (SD)	12.3 (5.4)	12.0 (4.8)	12.8 (6.5)	0.51^d^
**Age categories, years**				0.05
< 2	1 (0.7)	0 (0)	1 (2.3)	
2–4	8 (5.7)	3 (3.1)	5 (11.6)	
5–7	21 (15.0)	15 (15.5)	6 (14.0)	
8–12	55 (39.3)	43 (44.3)	12 (27.9)	
13–18	36 (25.7)	26 (26.8)	10 (23.3)	
> 18	19 (13.6)	10 (10.3)	9 (20.9)	
Age at diagnosis,^a^ years, mean (SD)	7.1 (5.0)	7.0 (4.6)	7.5 (5.7)	0.59^d^
**Child’s gender**^**b**^				0.46
Female	69 (49.3)	50 (51.6)	19 (44.2)	
Male	71 (50.7)	47 (48.5)	24 (55.8)	
**Time intervals since diagnosis at time of study**				0.03
During OT	15 (10.7)	10 (10.3)	5 (11.6)	
1st year after the end of OT	19 (13.6)	9 (9.3)	10 (23.3)	
Between 1 and 5 years after the end of OT	52 (37.1)	43 (44.3)	9 (20.9)	
≥ 5 years after the end of OT	50 (35.7)	33 (34.0)	17 (39.5)	
Other (recurrence or 2nd cancer)	4 (2.9)	2 (2.1)	2 (4.7)	
**Type of cancer**				0.51
Leukemia	42 (30.0)	30 (30.9)	12 (27.9)	
Malignant solid tumors	42 (30.0)	29 (30.0)	13 (30.2)	
Leukemia/malignant solid tumors	3 (2.1)	3 (3.1)	0 (0.0)	
Central nervous system (including retinoblastoma)	34 (24.3)	25 (25.8)	9 (20.9)	
Lymphoma	13 (9.3)	7 (7.2)	6 (14.0)	
Other	6 (4.3)	3 (3.1)	3 (7.0)	
**Treatment received (4 different binary variables)**				
Chemotherapy	106 (76.3)	73 (76.0)	33 (76.7)	0.64
Surgery	82 (58.6)	56 (57.7)	26 (60.5)	0.85
Radiotherapy	33 (23.6)	22 (22.7)	11 (25.6)	0.83
Stem cell transplantation	16 (11.4)	14 (14.4)	2 (4.7)	0.15
**CAM supplemental healthcare insurance**				0.004
Yes	102 (72.9)	78 (80.4)	24 (55.8)	
No	28 (20.0)	16 (16.5)	12 (27.9)	
Don’t know	10 (7.1)	3 (3.1)	7 (16.3)	

All values represent n (%) except where otherwise indicated.

*CAM* Complementary and alternative medicine, *OT* Oncology treatment

^a^Mean age (SD) at diagnosis of non-respondents (*n* = 278): 8.6 (5.5) years

^b^Gender of non-respondents (*n* = 278): female *n* = 124 (44.6%), male *n* = 154 (55.4%)

^c^Statistical analyses were conducted by using Fisher’s exact test, except where otherwise indicated

^d^The student t-test was used for age when considered continuous

### Prevalence of use of different types of CAM at specific time intervals

The prevalence of use and the distribution of CAMs used according to defined time intervals are summarized in Table 2. CAMs were used before diagnosis by 54.3% of patients; among these patients, six stopped using CAM after diagnosis. Since diagnosis, 69.3% of patients (95% confidence interval: 61.2–76.3) had used CAM, regardless of time intervals. During each time interval since diagnosis, between 50 and 58.8% of the patients used at least one CAM.

**Table 2:** Number of patients using complementary and alternative medicine modalities before and after diagnosis (multiple possible entries)

CAM therapies	Before diagnosis	After diagnosis			
	***n*** (%) (***n*** = 140)	During OT ***n*** (%) (***n*** = 140)	1st y after the end of OT ***n*** (%) (***n*** = 121)	Between 1 and 5 y after the end of OT ***n*** (%) (***n*** = 102)	≥5 y after the end of OT ***n*** (%) (***n*** = 50)
At least 1 of all modalities	76 (54.3)	77 (55.0)	63 (52.1)	60 (58.8)	25 (50.0)
[95% CI] (%)	[46.0–62.3]	[46.7–63.0]	[43.2–60.8]	[49.1–67.9]	[36.6–63.4]
Specific modalities					
Acupuncture	1 (0.7)	5 (3.6)	1 (0.8)	1 (1.0)	2 (4.0)
Aromatherapy	20 (14.3)	15 (10.7)	16 (13.2)	17 (16. 7)	10 (20.0)
Art therapy	1 (0.7)	5 (3.6)	1 (0.8)	1 (1.0)	0 (0.0)
Bioresonance	3 (2.1)	5 (3.6)	2 (1.7)	2 (2.0)	1 (2.0)
Bach flowers	27 (19.3)	14 (10.0)	13 (10.7)	18 (17.7)	8 (16.0)
Dietary supplements	27 (19.3)	18 (12.9)	21 (17.4)	19 (18.6)	8 (16.0)
Herbal medicine	2 (1.4)	6 (4.3)	3 (2.5)	4 (3.9)	2 (4.0)
Homeopathy	56 (40.0)	38 (27.1)	41 (33.9)	37 (36.3)	11 (22.0)
Hypnosis	2 (1.4)	18 (12.9)	3 (2.5)	6 (5.9)	3 (6.0)
Kinesiology^a^	17 (12.1)	6 (4.3)	6 (5.0)	9 (8.8)	3 (6.0)
Meditation	4 (2.9)	2 (1.4)	2 (1.7)	1 (1.0)	1 (2.0)
Music therapy	1 (0.7)	3 (2.1)	1 (0.8)	1 (1.0)	0 (0.0)
Osteopathy^b^	44 (31.4)	16 (11.4)	26 (21.5)	31 (30.4)	14 (28.0)
Reflexology	2 (1.4)	3 (2.1)	4 (3.3)	2 (2.0)	1 (2.0)
Shiatsu	1 (0.7)	1 (0.7)	1 (0.8)	1 (1.0)	1 (2.0)
Therapeutic massage	11 (7.9)	12 (8.6)	6 (5.0)	7 (6.9)	2 (4.0)
TCM^b^	1 (0.7)	1 (0.7)	1 (0.8)	0 (0.0)	1 (2.0)
Yoga	3 (2.1)	3 (2.1)	0 (0.0)	3 (2.9)	1 (2.0)
Traditional healer	1 (0.7)	9 (6.4)	5 (4.1)	2 (2.0)	2 (4.0)
Energy therapy	1 (0.7)	3 (2.1)	3 (2.5)	3 (2.9)	0 (0.0)
Other^c^	1 (0.7)	4 (2.9)	3 (2.5)	3 (2.9)	2 (4.0)

*CAM* Complementary and alternative medicine, *OT* Oncology treatment, *y* Years, *TCM* Traditional Chinese medicine.

^a^One respondent answered “I don’t remember” and one answered “I don’t wish to answer”

^b^One respondent answered “I don’t remember”

^c^Acupressure, anthroposophical medicine, Ayurveda, chromopuncture, naturopathy, orthobionomy, sophrology, and Tai chi were included

Homeopathy was used by more than 20% of patients during each time interval. Osteopathy was used by more than 20% of patients except during oncology treatment (11.4%). More than 10% of patients used aromatherapy, Bach flowers or dietary supplements during each time interval.

**Fig. 2:**
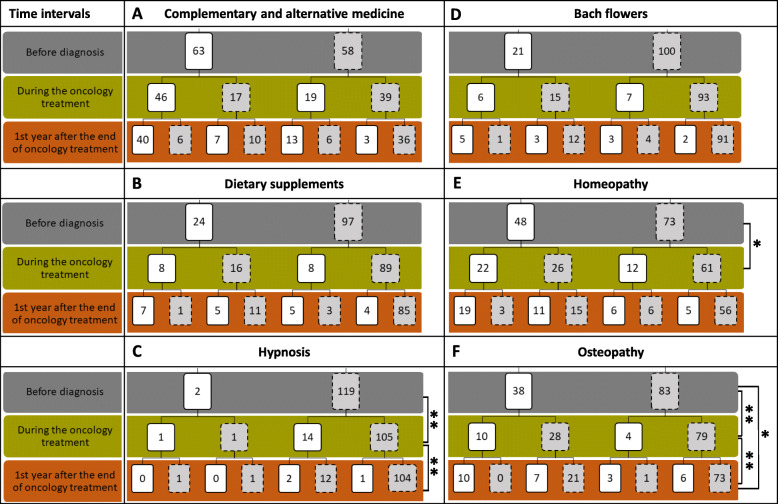
Number of patients using complementary and alternative medicine according to oncology time intervals. Results are expressed in terms of number of respondents. Total of respondents for each complementary and alternative medicine *n* = 121. White boxes represent complementary and alternative medicine users and grey boxes, non-users. *Significant at *p* < 0.05; **significant at *p* < 0.005

In Fig. 2, we describe in more detail the prevalence of CAM use in general and that of the five most often used CAMs before diagnosis, during oncology treatment, and during the first year after the end of oncology treatment. To compare data, we analyzed the statistics for 121 respondents who were at least in the first year after the end of oncology treatment. With all modalities combined, 40 patients used CAM throughout these three time. Nineteen patients began to use CAM during oncology treatment and 13 continued thereafter. If patients used CAM before diagnosis, the great majority continued to use it during oncology treatment, as well as during the first year after the end of oncology treatment, although not necessarily the same modality. Indeed, when we considered the five most often used types of CAM individually (Bach flowers, dietary supplements, homeopathy, hypnosis, and osteopathy), the tendency changed: among patients who used Bach flowers or dietary supplements before diagnosis, most of them stopped during oncology treatment and did not resume afterward. Osteopathy was more likely to be used before diagnosis than it was during oncology treatment (*p* < 0.005), and more likely to be used during the first year after the end of oncology treatment than it was during oncology treatment (*p* < 0.005). Concerning hypnosis, its use was higher during oncology treatment than it was before diagnosis (*p* < 0.005) or during the year after the end of oncology treatment (*p* < 0.005). Homeopathy was more likely to be used before diagnosis than it was during oncology treatment (*p* < 0.05).

### Sources of information on CAM use

The information sources most often consulted by respondents were friends or family, as well as media (Table 3). Forty-four percent of all respondents would like to have access to a specialized service for CAM counseling and treatment.

**Table 3:** Source of information about complementary and alternative medicine (multiple entries possible)

Source of information	Total		CAM users		CAM non-users		*p*-value (Fisher)
	*N*_total_^a^	Often consulted, *n* (%)	*N*_*t*otal_^a^	Often consulted, *n* (%)	*N*_total_^a^	Often consulted, *n* (%)	
Friends or family	137	36 (26.3)	97	29 (29.9)	40	7 (17.5)	0.20
Media *(Internet, newspaper, book, TV, radio, advertising)*	139	34 (24.5)	97	23 (23.7)	42	11 (26.2)	0.83
Medical specialist in CAM *(homeopath, acupuncturist, hypnotist)*	138	32 (23.2)	96	28 (29.2)	42	4 (9.5)	0.01
Therapist in CAM *(non-physician)*	138	32 (23.2)	96	30 (31.3)	42	2 (4.8)	0.00
Community pharmacist	138	24 (17.4)	96	21 (21.9)	42	3 (7.1)	0.05
Physician *(family, general practitioner)*	137	18 (13.1)	95	13 (13.7)	42	5 (11.9)	0.99
Pediatric oncologist	138	15 (10.9)	96	10 (10.4)	42	5 (11.9)	0.77
Oncology nurse	138	14 (10.1)	96	9 (9.4)	42	5 (11.9)	0.76
Parents’ association	138	8 (5.8)	96	5 (5.2)	42	3 (7.1)	0.70

*CAM* Complementary and alternative medicine.

^a^The *N*_total_ number changes because the answers “I do not wish to answer” are not shown

### Communication and perceptions about CAM use

When asked about communication with the pediatric oncologist concerning CAM use, 43.3% (*n* = 42/97) of CAM users and 27.9% (*n* = 12/43) of non-users stated that they had such a discussion. In most cases (87.0% *n* = 47/54), one of the parents began the discussion (90.5% (*n* = 38/42) of CAM users; 75.0% (*n* = 9/12) of non-users) and in 14.8% (*n* = 8/54) of cases, the oncologist did. The oncologist did not suggest using CAM in 78.6% (*n* = 110/140) of the cases. Had the oncologist proposed CAM use, 74.3% (*n* = 104/140) of the respondents (83.5% (*n* = 81/97) of CAM users; 53.5% (*n* = 23/43) of non-users, *p* < 0.005) would have tried it for their child in the context of the cancer, 7.9% (*n* = 11/140) did not know, and 5.0% (*n* = 7/140) would not have tried it.

Moreover, when considering CAM in general, 39.2% of CAM users and 30.2% of non-users agreed that CAM might interact with oncology treatments, and 23.7% of CAM users and 44.2% of non-users did not know. However, when considering herbal treatment specifically, that number increased among CAM users as half of them agreed that the use of herbal treatments presented interaction risks with oncology treatment (Table 4).

**Table 4:** Perception of complementary and alternative medicine use by patient’s parents

Question^**a**^	Total ***n*** (%) (***n*** = 140)	CAM users ***n*** (%) (***n*** = 97)	CAM non-users ***n*** (%) (***n*** = 43)	***p***-value (t-test)
**1.** *“I received enough information from the oncologist about CAM benefits.”*				0.46
Disagree	91 (65.0)	67 (69.1)	24 (55.8)	
Neither agree nor disagree	19 (13.6)	13 (13.4)	6 (14.0)	
Agree	14 (10.0)	8 (8.3)	6 (14.0)	
I do not know	12 (8.6)	7 (7.2)	5 (11.6)	
I do not wish to respond	4 (2.9)	2 (2.1)	2 (4.7)	
**2.** *“I received enough information from the oncologist about CAM risks.”*				0.45
Disagree	70 (50.0)	48 (49.5)	22 (51.2)	
Neither agree nor disagree	26 (18.6)	21 (21.7)	5 (11.6)	
Agree	29 (20.7)	20 (20.6)	9 (20.9)	
I do not know	11 (7.9)	6 (6.2)	5 (11.6)	
I do not wish to respond	4 (2.9)	2 (2.1)	2 (4.7)	
**3.** *“Using CAM may interact with my child’s conventional treatment.”*				0.09
Disagree	24 (17.1)	18 (18.6)	6 (14.0)	
Neither agree nor disagree	19 (13.6)	16 (16.5)	3 (7.0)	
Agree	51 (36.4)	38 (39.2)	13 (30.2)	
I do not know	42 (30.0)	23 (23.7)	19 (44.2)	
I do not wish to respond	4 (2.9)	2 (2.1)	2 (4.7)	
**4.** *“Using herbal treatments may interact with my child’s conventional treatment.”*				0.23
Disagree	19 (13.6)	13 (13.4)	6 (14.0)	
Neither agree nor disagree	15 (10.7)	12 (12.4)	3 (7.0)	
Agree	65 (46.4)	49 (50.5)	16 (37.2)	
I do not know	37 (26.4)	21 (21.7)	16 (37.2)	
I do not wish to respond	4 (2.9)	2 (2.1)	2 (4.7)	

*CAM* Complementary and alternative medicine.

^a^The categories “strongly agree” and “agree” were grouped into “agree” and the categories “strongly disagree” and “disagree” were grouped into “disagree”

Among CAM users who received the online questions about CAM disclosure (*n* = 65), 60.0% told the oncologist about their child’s CAM use, 15.4% did not remember, and one person did not wish to respond. The oncologist’s reaction to CAM disclosure was to encourage the respondent to continue CAM use (41.0%), to give the respondent information and explanations concerning contraindications (33.3%), or to make no recommendation (25.6%). In one case, the oncologist asked the respondent to stop using it. In contrast, 23.1% of CAM users did not tell the oncologist about CAM use because “he/she did not ask” (53.3%), “it does not matter if I tell him/her that” (40.0%), or “he/she would not have understood” (20.0%).

## Discussion

### Prevalence

Our results showed a higher prevalence of CAM use since diagnosis than in most studies included in a 2010 systematic review [1] or published since then [2, 11–13, 23]. Indeed, our prevalence is higher than the only Swiss study published that included pediatric oncology patients [2], than that of the Swiss adult population [22], Swiss patients with cancer [22, 27], as well as than that of Swiss [28] or European children [3]. Half of our respondents used CAM before diagnosis. Similar results were reported in two studies that investigated this time interval [2, 23]. The prevalence of CAM use could be influenced by subscription to a specific CAM supplemental healthcare insurance. In our study, most CAM users and more than half of non-users had subscribed to one. In 2012, 59.9% of the Swiss adult population had CAM supplemental health insurance coverage [22]. A tendency has been reported for persons with CAM supplemental healthcare insurance to use CAM more frequently than is the case for those without such insurance [22, 29]. However, subscription to CAM supplemental healthcare insurance is usually denied to patients after a cancer diagnosis, even children. Given the high percentage of respondents with such healthcare insurance, we assume that they took it out before the cancer diagnosis.

### Time intervals

Precise post-diagnosis time intervals are rare in previously published studies [7, 10, 12, 13]. Only one study investigated CAM use according to such specific time intervals [13]. However, our results are difficult to compare with those of the latter because that study grouped CAM in categories and did not analyze them separately. Our results, which show that most of our patients used CAM between 1 and 5 years after the end of oncology treatment, are consistent with those of Ndao et al. [13] but not with those of Längler et al., which highlighted that CAM was mostly used concomitantly with oncology treatment [7]. Notably, only one third of our respondents had yet reached the time interval of ≥5 years after the end of oncology treatment. Therefore, we could not anticipate the prevalence of CAM use had all of our respondents met this time interval.

### Modalities of CAM according to time intervals

Considering all modalities, if patients used CAM before diagnosis, the great majority would continue to use CAM during oncology treatment, as well as during the first year after the end of oncology treatment, although not necessarily the same modality. However, when considering the five most used modalities individually, a different tendency of use was observed among patients who were already using them before diagnosis: most patients stopped during oncology treatment and did not resume afterward (Bach flowers, dietary supplement, and osteopathy). This was not the case for hypnosis. The higher use of hypnosis during oncology treatment than before diagnosis is likely due to the integration of hypnosis in the hematology-oncology unit 20 years ago to cope with pain, especially procedural pain (lumbar puncture, central venous access device, etc.). Hypnosis was the only CAM listed in our questionnaire to which patients had access inside the unit. The decrease in hypnosis use after the patients had completed oncology treatment could be linked with the loss of care offer. Our results highlight that osteopathy use was higher before diagnosis and during the first year after the end of oncology treatment than during oncology treatment. We hypothesize that osteopathy is less used during oncology treatment because of possible adverse events [30] linked with thrombocytopenia and risk of bleeding. However, others have shown that use of manipulative and body-based therapies (including osteopathy) was higher during treatment and during 0–4 years after treatment than during prediagnosis [13]. Some CAM types may have been stopped because of preoccupation with interaction risks between CAM and oncology treatment, as around a third of CAM users in our study were aware of such potential interaction risks when considering CAM in general, and one half when considering herbal treatments specifically.

### Source of information

Friends or family, as well as media, were the most consulted sources of information about CAM, a result in line with the trend reported by others [7, 9, 11, 23, 31, 32]. In other studies, most respondents considered that CAM information should be provided by pediatric oncologists [8, 32], who also consider it to be their role [33]. Where integrative oncology programs exist, more than half of the respondents would use them [13]. We hypothesize that respondents were less likely to consult the pediatric oncologist as a source of information because he or she did not ask about CAM use [34].

### Communication between the pediatric oncologist and the patient’s parents

Among respondents who discussed CAM use with the oncologist, most of them initiated the discussion, as also suggested by Singendonk et al. [8]. Around two thirds of CAM users disclosed their CAM use to the oncologist. This result falls in the range of published percentages of CAM use disclosure to the oncologist [2, 7, 8, 10, 13, 31]. One quarter of our disclosing respondents stated that their oncologist did not make any recommendation regarding the continuation, discontinuation or safety of CAM use. This absence of recommendation and information could be due to a lack of time or knowledge about CAM [18, 35] or a lack of specific CAM training during medical school [36].

Oncologists should discuss and inform their patients about CAM use to warn them about interaction risks [32]. Being first-line providers, they are key actors in identifying potential benefits and risks for patients. Should oncologists lack knowledge concerning CAM use, they could redirect patients to qualified integrative physicians within the hospital as has been suggested in Ben-Arye et al. [37]. This approach is all the more important because, more than a quarter of our respondents did not know whether, and more than one tenth disagreed with the position that, the use of herbal treatments presented interaction risks with oncology treatment. Moreover, although CAM integration in pediatric oncology wards is rare [38–42], it is important to parents that CAM be offered in the hospital [8]. From the point of view of pediatric oncologists, some CAM types, such as meditation, yoga, and acupuncture, could improve the quality of life of their patients, whereas others, such as dietary supplements and herbal medicines, could potentially be harmful [18]. CAM in the form of music therapy was not routinely used in our study, although an effect on anxiety and depression in adult cancer patients has been shown [43]. However, a recent systematic review did not identify any CAM that could be recommended in a curative intent [44].

Furthermore, during each time interval since diagnosis, between 50 and 60% of our patients used CAM. Therefore, discussion about CAM use needs to take place several times during the oncology follow-up because the risks of its use might differ during these time intervals and patients might use different CAM types during the pathway of the disease.

The survey was conducted in a single academic hospital and therefore cannot be representative of other hospitals. Respondents’ interest in or use of CAM could also be considered a source of selection bias because of their potentially higher interest in the subject, leading to an underrepresentation of CAM non-users among respondents, which could explain the low participation rate in this study. This low rate could also be due to (1) the period in which the questionnaire was sent, which was just before summer vacation; (2) families living with or having experienced childhood cancer being often asked to participate in surveys; and (3) the difficulty in revisiting painful memories. Only 10% of patients still under oncology treatment participated in this study. The small representation of this group could be due to lack of time and stress during this time interval because of the new family organization adapted to oncology treatment planning [45], and the emotional burden. However, patients in the respondents’ and the non-respondents’ group were comparable, as there were no significant differences (gender, type of cancer, treatment received) except for age at the time of the study and at diagnosis. Moreover, the frequency of CAM use was not investigated, and so it is impossible to know whether patients used CAM frequently or not. We selected patients who received a cancer diagnosis within a 10-year period that could increase the risk of recall bias. Reasons of CAM use before and after diagnosis were not investigated. Finally, as hypnosis was the only CAM proposed to patients in the pediatric hematology-oncology unit during oncology treatment, we could not determine whether its use was patient-initiated or resulted from its availability in the unit.

## Conclusions

The high prevalence of CAM use and the different trends of use during the oncology care pathway and afterward underline the need to increase open discussion about CAM in the pediatric oncology setting, all the more so because some therapies have proven to be useful in the treatment of oncology treatment side effects, whereas others could potentially be harmful. Oncologist information and training on CAM is required to improve communication. Further investigations are needed, however, to confirm our results with a larger sample.

## Supplementary Information

**Additional file 1.** Questionnaire This file contains the questionnaire of our study in English.

**Additional file 2.** Questionnaire This file contains the questionnaire of our study in French.

## Data Availability

The datasets generated and/or analyzed during the current study are not publicly available due to privacy or ethical restrictions but are available from the corresponding author on reasonable request.

## Abbreviations

CAM: Complementary and alternative medicine
