# Supplementary material for: Complementary and alternative medicine use by pediatric oncology patients before, during, and after treatment
Source: BMC Complement Med Ther. 2021 Mar 18;21:96. doi: 10.1186/s12906-021-03271-9 (PMC7977159; doi:10.1186/s12906-021-03271-9)
Supplement: Supplementary file 1 — Additional file 1. Questionnaire This file contains the questionnaire of our study in English. [file 12906_2021_3271_MOESM1_ESM.pdf]

Additional Material

Questionnaire

Part A: Complementary medicines and your child

Definitions used in this questionnaire:

Complementary medicine (also known as alternative medicine, parallel medicine, natural medicine, etc.): complementary medicine refers to a broad set of healthcare practices that are not part of that country's own tradition or conventional medicine and are not fully integrated into the dominant healthcare system.

Here are some examples of complementary medicine:

acupuncture	hypnosis	sophrology
anthroposophic medicine	kinesiology	tai chi
aromatherapy (essential oils)	meditation	therapeutic massage
art therapy	osteopathy	traditional Chinese medicine
herbal medicine	reflexology	yoga
homeopathy	shiatsu	

Conventional treatment: conventional treatment includes therapies such as chemotherapy, radiotherapy, stem cell transplantation, surgery, and immunotherapy.

Note: In this questionnaire, the term "complementary medicines" will always be used in the plural but your answers may refer to only one complementary medicine.

A1. Currently, which age group is your child in?

- <2 years old ☐
- 2-4 years old ☐
- 5-7 years old ☐
- 8-12 years old ☐
- 13-18 years old ☐
- >18 years old ☐

A2. Since his/her birth, has your child ever used complementary medicines that are related or not related to his/her oncological disease? If yes, specify which one from the list below. If no, tick the first box "NONE". Please do not take into account complementary medicines provided by a healthcare provider of the CHUV during your stay.

- NONE ☐
- Acupuncture ☐
- Aromatherapy (essential oils) ☐
- Art therapy ☐
- Ayurveda ☐
- Bioresonance ☐
- Dietary supplements (vitamins, trace elements) ☐
- Bach flowers ☐
- Homeopathy ☐
- Hypnosis ☐
- Kinesiology ☐
- Therapeutic massage ☐
- Anthroposophic medicine (including mistletoe therapy) ☐
- Traditional Chinese medicine (except acupuncture) ☐

Meditation ☐

Music therapy ☐

Osteopathy ☐

Herbal medicine ☐

Reflexology ☐

Sophrology ☐

Shiatsu ☐

Tai chi ☐

Yoga ☐

Other ☐

A3. Please write the name(s) of the other complementary medicines used below:

complementary medicine 1 ☐

complementary medicine 2 ☐

complementary medicine 3 ☐

Part B: Complementary medicines and your child's oncology disease

B1. Does your child use complementary medicines <u>in relation to his/her oncology disease</u>? Here is a reminder of some complementary medicines:

Acupuncture	Massage thérapeutique	Réflexologie
Aromathérapie (Huiles essentielles)	Médecine anthroposophique	Shiatsu
Art-thérapie	Médecine traditionnelle chinoise	Sophrologie
Homéopathie	Méditation	Tai chi
Hypnose	Ostéopathie	Yoga
Kinésiologie	Phytothérapie (thérapie par les plantes)	...

☐ Yes

☐ No

☐ I don't wish to answer

Part C: Complementary medicine use depending on oncology disease time intervals

C1. <u>Currently</u>, which of the following time intervals related to oncology disease is your child in?

If your child is in a relapse period, please check the "Other" box

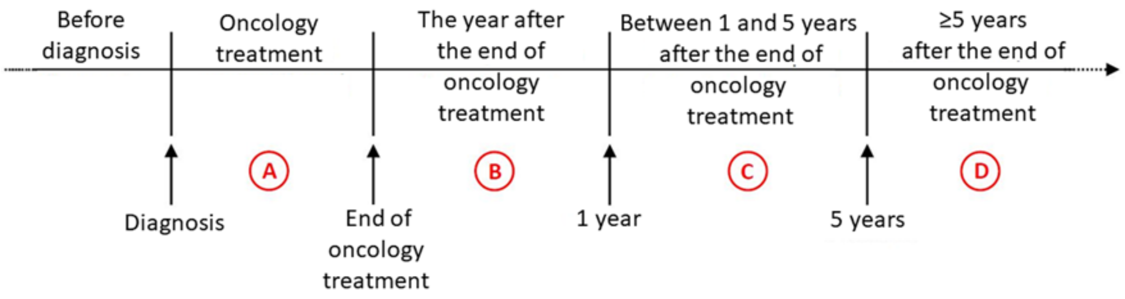

A ☐

B ☐

C ☐

D ☐

Other ☐

C2. If other, please specify which of the following two possibilities:

Information: The treatment of certain childhood cancers carries the risk of developing another oncology disease. Do not hesitate to talk to your oncologist about it.

Relapse ☐

Diagnosis of another oncology disease ☐

C3. For each complementary medicine used, please indicate when your child used it.

	Before diagnosis	During oncology treatment	1 year after the end of oncology treatment	1 to 5 years after the end of oncology treatment	≥5 years after the end of oncology treatment	I don't remember	I don't wish to answer
**Acupuncture**	☐	☐	☐	☐	☐	☐	☐
**Anthroposophic medicine**	☐	☐	☐	☐	☐	☐	☐
**Aromatherapy**	☐	☐	☐	☐	☐	☐	☐
**Art therapy**	☐	☐	☐	☐	☐	☐	☐
**Ayurveda**	☐	☐	☐	☐	☐	☐	☐
**Bioresonance**	☐	☐	☐	☐	☐	☐	☐
**Bach flowers**	☐	☐	☐	☐	☐	☐	☐
**Dietary supplements**	☐	☐	☐	☐	☐	☐	☐
**Herbal medicine**	☐	☐	☐	☐	☐	☐	☐
**Homeopathy**	☐	☐	☐	☐	☐	☐	☐
**Hypnosis**	☐	☐	☐	☐	☐	☐	☐
**Kinesiology**	☐	☐	☐	☐	☐	☐	☐
**Meditation**	☐	☐	☐	☐	☐	☐	☐
**Music therapy**	☐	☐	☐	☐	☐	☐	☐
**Osteopathy**	☐	☐	☐	☐	☐	☐	☐
**Reflexology**	☐	☐	☐	☐	☐	☐	☐
**Shiatsu**	☐	☐	☐	☐	☐	☐	☐
**Sophrology**	☐	☐	☐	☐	☐	☐	☐
**Tai chi**	☐	☐	☐	☐	☐	☐	☐
**Therapeutic massage**	☐	☐	☐	☐	☐	☐	☐
**Traditional Chinese medicine**	☐	☐	☐	☐	☐	☐	☐
**Yoga**	☐	☐	☐	☐	☐	☐	☐
**Other 1**	☐	☐	☐	☐	☐	☐	☐
**Other 2**	☐	☐	☐	☐	☐	☐	☐
**Other 3**	☐	☐	☐	☐	☐	☐	☐

C3a. For each complementary medicine used, please indicate <u>when</u> your child used it <u>before the relapse</u>.

	Not used before relapse	Before diagnosis	During oncology treatment	1 year after the end of oncology treatment	1 to 5 years after the end of oncology treatment	≥5 years after the end of oncology treatment	I don't remember	I don't wish to answer
**Acupuncture**	☐	☐	☐	☐	☐	☐	☐	☐
**Anthroposophic medicine**	☐	☐	☐	☐	☐	☐	☐	☐
**Aromatherapy**	☐	☐	☐	☐	☐	☐	☐	☐
**Art therapy**	☐	☐	☐	☐	☐	☐	☐	☐
**Ayurveda**	☐	☐	☐	☐	☐	☐	☐	☐
**Bioresonance**	☐	☐	☐	☐	☐	☐	☐	☐
**Bach flowers**	☐	☐	☐	☐	☐	☐	☐	☐
**Dietary supplements**	☐	☐	☐	☐	☐	☐	☐	☐
**Herbal medicine**	☐	☐	☐	☐	☐	☐	☐	☐
**Homeopathy**	☐	☐	☐	☐	☐	☐	☐	☐
**Hypnosis**	☐	☐	☐	☐	☐	☐	☐	☐
**Kinesiology**	☐	☐	☐	☐	☐	☐	☐	☐
**Meditation**	☐	☐	☐	☐	☐	☐	☐	☐
**Music therapy**	☐	☐	☐	☐	☐	☐	☐	☐
**Osteopathy**	☐	☐	☐	☐	☐	☐	☐	☐
**Reflexology**	☐	☐	☐	☐	☐	☐	☐	☐
**Shiatsu**	☐	☐	☐	☐	☐	☐	☐	☐
**Sophrology**	☐	☐	☐	☐	☐	☐	☐	☐
**Tai chi**	☐	☐	☐	☐	☐	☐	☐	☐
**Therapeutic massage**	☐	☐	☐	☐	☐	☐	☐	☐
**Traditional Chinese medicine**	☐	☐	☐	☐	☐	☐	☐	☐
**Yoga**	☐	☐	☐	☐	☐	☐	☐	☐
**Other 1**	☐	☐	☐	☐	☐	☐	☐	☐
**Other 2**	☐	☐	☐	☐	☐	☐	☐	☐
**Other 3**	☐	☐	☐	☐	☐	☐	☐	☐

	C3b. For each complementary medicine used, please indicate **when** your child used it **after the relapse**.							
	Not used after relapse	Before diagnosis	During oncology treatment	1 year after the end of oncology treatment	1 to 5 years after the end of oncology treatment	≥5 years after the end of oncology treatment	I don't remember	I don't wish to answer
**Acupuncture**	☐	☐	☐	☐	☐	☐	☐	☐
**Anthroposophic medicine**	☐	☐	☐	☐	☐	☐	☐	☐
**Aromatherapy**	☐	☐	☐	☐	☐	☐	☐	☐
**Art therapy**	☐	☐	☐	☐	☐	☐	☐	☐
**Ayurveda**	☐	☐	☐	☐	☐	☐	☐	☐
**Bioresonance**	☐	☐	☐	☐	☐	☐	☐	☐
**Bach flowers**	☐	☐	☐	☐	☐	☐	☐	☐
**Dietary supplements**	☐	☐	☐	☐	☐	☐	☐	☐
**Herbal medicine**	☐	☐	☐	☐	☐	☐	☐	☐
**Homeopathy**	☐	☐	☐	☐	☐	☐	☐	☐
**Hypnosis**	☐	☐	☐	☐	☐	☐	☐	☐
**Kinesiology**	☐	☐	☐	☐	☐	☐	☐	☐
**Meditation**	☐	☐	☐	☐	☐	☐	☐	☐
**Music therapy**	☐	☐	☐	☐	☐	☐	☐	☐
**Osteopathy**	☐	☐	☐	☐	☐	☐	☐	☐
**Reflexology**	☐	☐	☐	☐	☐	☐	☐	☐
**Shiatsu**	☐	☐	☐	☐	☐	☐	☐	☐
**Sophrology**	☐	☐	☐	☐	☐	☐	☐	☐
**Tai chi**	☐	☐	☐	☐	☐	☐	☐	☐
**Therapeutic massage**	☐	☐	☐	☐	☐	☐	☐	☐
**Traditional Chinese medicine**	☐	☐	☐	☐	☐	☐	☐	☐
**Yoga**	☐	☐	☐	☐	☐	☐	☐	☐
**Other 1**	☐	☐	☐	☐	☐	☐	☐	☐
**Other 2**	☐	☐	☐	☐	☐	☐	☐	☐
**Other 3**	☐	☐	☐	☐	☐	☐	☐	☐

	C3c. For each complementary medicine used, please indicate **when** your child used it **before diagnosis of another oncology disease**.							
	Not used before diagnosis of another oncology disease	Before diagnosis	During oncology treatment	1 year after the end of oncology treatment	1 to 5 years after the end of oncology treatment	≥5 years after the end of oncology treatment	I don't remember	I don't wish to answer
**Acupuncture**	☐	☐	☐	☐	☐	☐	☐	☐
**Anthroposophic medicine**	☐	☐	☐	☐	☐	☐	☐	☐
**Aromatherapy**	☐	☐	☐	☐	☐	☐	☐	☐
**Art therapy**	☐	☐	☐	☐	☐	☐	☐	☐
**Ayurveda**	☐	☐	☐	☐	☐	☐	☐	☐
**Bioresonance**	☐	☐	☐	☐	☐	☐	☐	☐
**Bach flowers**	☐	☐	☐	☐	☐	☐	☐	☐
**Dietary supplements**	☐	☐	☐	☐	☐	☐	☐	☐
**Herbal medicine**	☐	☐	☐	☐	☐	☐	☐	☐
**Homeopathy**	☐	☐	☐	☐	☐	☐	☐	☐
**Hypnosis**	☐	☐	☐	☐	☐	☐	☐	☐
**Kinesiology**	☐	☐	☐	☐	☐	☐	☐	☐
**Meditation**	☐	☐	☐	☐	☐	☐	☐	☐
**Music therapy**	☐	☐	☐	☐	☐	☐	☐	☐
**Osteopathy**	☐	☐	☐	☐	☐	☐	☐	☐
**Reflexology**	☐	☐	☐	☐	☐	☐	☐	☐
**Shiatsu**	☐	☐	☐	☐	☐	☐	☐	☐
**Sophrology**	☐	☐	☐	☐	☐	☐	☐	☐
**Tai chi**	☐	☐	☐	☐	☐	☐	☐	☐
**Therapeutic massage**	☐	☐	☐	☐	☐	☐	☐	☐
**Traditional Chinese medicine**	☐	☐	☐	☐	☐	☐	☐	☐
**Yoga**	☐	☐	☐	☐	☐	☐	☐	☐
**Other 1**	☐	☐	☐	☐	☐	☐	☐	☐
**Other 2**	☐	☐	☐	☐	☐	☐	☐	☐
**Other 3**	☐	☐	☐	☐	☐	☐	☐	☐

	C3d. For each complementary medicine used, please indicate **when** your child used it **after diagnosis of another oncology disease**.							
	Not used after diagnosis of another oncology disease	Before diagnosis	During oncology treatment	1 year after the end of oncology treatment	1 to 5 years after the end of oncology treatment	≥5 years after the end of oncology treatment	I don't remember	I don't wish to answer
**Acupuncture**	☐	☐	☐	☐	☐	☐	☐	☐
**Anthroposophic medicine**	☐	☐	☐	☐	☐	☐	☐	☐
**Aromatherapy**	☐	☐	☐	☐	☐	☐	☐	☐
**Art therapy**	☐	☐	☐	☐	☐	☐	☐	☐
**Ayurveda**	☐	☐	☐	☐	☐	☐	☐	☐
**Bioresonance**	☐	☐	☐	☐	☐	☐	☐	☐
**Bach flowers**	☐	☐	☐	☐	☐	☐	☐	☐
**Dietary supplements**	☐	☐	☐	☐	☐	☐	☐	☐
**Herbal medicine**	☐	☐	☐	☐	☐	☐	☐	☐
**Homeopathy**	☐	☐	☐	☐	☐	☐	☐	☐
**Hypnosis**	☐	☐	☐	☐	☐	☐	☐	☐
**Kinesiology**	☐	☐	☐	☐	☐	☐	☐	☐
**Meditation**	☐	☐	☐	☐	☐	☐	☐	☐
**Music therapy**	☐	☐	☐	☐	☐	☐	☐	☐
**Osteopathy**	☐	☐	☐	☐	☐	☐	☐	☐
**Reflexology**	☐	☐	☐	☐	☐	☐	☐	☐
**Shiatsu**	☐	☐	☐	☐	☐	☐	☐	☐
**Sophrology**	☐	☐	☐	☐	☐	☐	☐	☐
**Tai chi**	☐	☐	☐	☐	☐	☐	☐	☐
**Therapeutic massage**	☐	☐	☐	☐	☐	☐	☐	☐
**Traditional Chinese medicine**	☐	☐	☐	☐	☐	☐	☐	☐
**Yoga**	☐	☐	☐	☐	☐	☐	☐	☐
**Other 1**	☐	☐	☐	☐	☐	☐	☐	☐
**Other 2**	☐	☐	☐	☐	☐	☐	☐	☐
**Other 3**	☐	☐	☐	☐	☐	☐	☐	☐

C4. <u>Currently</u>, which oncology treatment(s) does your child receive?

- Chemotherapy ☐
- Immunotherapy ☐
- Radiotherapy ☐
- Surgery ☐
- Stem cell transplantation ☐
- Other ☐

Other

Part D: Complementary medicines and the oncologist in charge of your child

D1. During an appointment, did you <u>discuss complementary medicines</u> with the oncologist?

Yes ☐

No ☐

I don't remember ☐

I don't wish to answer ☐

D2. Who started this discussion <u>about complementary medicines</u>?

Me (the mother and/or the father) ☐

Pediatric oncologist ☐

My child ☐

Other ☐

Other

D3. Is the oncologist aware that you are using complementary medicines for your child <u>in relation to his/her oncology disease</u>?

Yes ☐

No ☐

I don't remember ☐

I don't wish to answer ☐

D4. How did <u>the oncologist</u> react when you told him/her that you were using complementary medicines for your child?

He/she encouraged me to continue the complementary medicine treatment. ☐

He/she asked me to stop the complementary medicine treatment. ☐

He/she provided me with information and explained the contraindication(s). ☐

He/she did not make a recommendation. ☐

He/she did not want to talk about it. ☐

Other ☐

Other

D5. Why has the oncologist not been informed that you were using complementary medicines for your child?

Because he/she did not ask me about it. ☐

Because he/she would not have understood. ☐

Because he/she would not have been happy about it. ☐

Because I forgot to mention it. ☐

Because it does not matter if I tell him/her that. ☐

Other ☐

Other

D6. Has the oncologist <u>suggested</u> that you use complementary medicines for your child <u>in relation to his/her oncology disease</u>?

Yes ☐

No ☐

I don't remember ☐

I don't wish to answer ☐

D7. Would you like to have access to a specialized consultation for complementary medicine advice and care at the CHUV for your child in relation to his/her oncology disease?

Yes ☐

No ☐

I don't know ☐

I don't wish to answer ☐

D8. For each of the sentences below (a-e), please indicate the answer that best represents <u>your opinion</u>.

	Strongly disagree	Disagree	Neither agree nor disagree	Agree	Strongly Agree	I don't remember	I don't wish to answer
**a.** I would be willing to try a complementary medicine method proposed by the oncologist in addition to the conventional treatment for my child.	☐	☐	☐	☐	☐	☐	☐
**b.** I have received enough information from the oncologist about the benefits of complementary medicines.	☐	☐	☐	☐	☐	☐	☐
**c.** I have received enough information from the oncologist about the risks of complementary medicines.	☐	☐	☐	☐	☐	☐	☐
**d.** Using complementary medicines may interact with the conventional treatment of my child.	☐	☐	☐	☐	☐	☐	☐
**e.** Using herbal medicines and/or treatments may interact with my child's conventional treatment.	☐	☐	☐	☐	☐	☐	☐

Part E: Sources of information used to inquire about complementary medicines

E1. In the list below, please indicate <u>the way(s)</u> you used to learn more about complementary medicines. Specify whether you consulted it/them a "<u>little</u>" or "<u>often</u>."

**Complementary medicine practitioners (non-physicians) include non-physician homeopaths, osteopaths, naturopaths, etc.*

	Not consulted	A little consulted	Often consulted	I don't wish to answer
Friends or family	☐	☐	☐	☐
Parents association	☐	☐	☐	☐
Pediatric oncology nurse	☐	☐	☐	☐
Physician specialized in pediatric oncology	☐	☐	☐	☐
Physician (GP, family medicine, etc.)	☐	☐	☐	☐
Physician specialized in complementary medicines (homeopath, acupuncturist, hypnotherapist, etc.)	☐	☐	☐	☐
Media (Internet, newspaper, book, TV, radio, advertising)	☐	☐	☐	☐
Pharmacist	☐	☐	☐	☐
Complementary medicine practitioner (non-physician)*	☐	☐	☐	☐

Part F: General information

F1. Who completed this questionnaire?

- The mother ☐
- The father ☐
- The child ☐
- Another family member ☐
- Other ☐

F2. Does your child have <u>supplemental healthcare insurance</u> for <u>complementary medicines</u>?

- Yes ☐
- No ☐
- I don’t know ☐
- I don’t wish to answer ☐

F3. Why doesn't your child have <u>supplemental healthcare insurance</u> for <u>complementary medicines</u>?

- Supplemental healthcare insurance for complementary medicines has been denied by the insurance company. ☐
- I do not have the necessary financial resources. ☐
- I do not know this type of supplemental healthcare insurance for complementary medicines. ☐
- I do not wish to take out such insurance for my child. ☐
- I don’t know. ☐
- Other ☐

Other

Part G: Finally ...

The last two questions below allow you to express your opinion on complementary medicines in general and on the questionnaire.

To finish the questionnaire, please click on the "Send" button at the bottom of the page.

G1. Do you have any comments on the subject of complementary medicines?

G2. Do you have any comments on this questionnaire?

G3. Would you like to be informed of the results of our study?

The results of this study will be available in a few months, after data analysis is completed. They will also be the subject of a publication in a scientific journal.

Yes ☐

No ☐

G4. Please write your email address below so that we can contact you if you would like to be informed of the results.

Your email address will only be used to send you the results of our study. The transmission of your email address does not in any way affect the confidentiality of your answers.
