# Supplementary material for: Complementary and alternative medicine use by pediatric oncology patients before, during, and after treatment
Source: BMC Complement Med Ther. 2021 Mar 18;21:96. doi: 10.1186/s12906-021-03271-9 (PMC7977159; doi:10.1186/s12906-021-03271-9)
Supplement: Supplementary file 2 — Additional file 2. Questionnaire This file contains the questionnaire of our study in French. [file 12906_2021_3271_MOESM2_ESM.pdf]

Additional Material

Questionnaire

Informations:

* Ce questionnaire est destiné à être rempli par le-s parent-s du patient en hémato- oncologie pédiatrique. Votre enfant peut bien entendu vous aider à le compléter.

* Il faut compter environ 25 minutes pour remplir le questionnaire.

* Répondez aux questions le plus spontanément possible : il n'y a pas de bonnes ou de mauvaises réponses, seul votre avis compte.

* Vos réponses seront traitées de manière confidentielle et ne seront pas transmises au médecin prenant en charge votre enfant.

* Merci de répondre à toutes les questions : un questionnaire incomplet est difficilement utilisable.

Part A: Les médecines complémentaires et votre enfant

Définitions utilisées dans ce questionnaire :

Médecines complémentaires (appelées aussi médecines alternatives, parallèles, douces, naturelles, etc.) : les médecines complémentaires sont définies comme un vaste ensemble de pratiques de soins de santé qui n'appartiennent pas à la tradition du pays et ne sont pas intégrées dans le système de santé dominant.

Voici quelques exemples de médecines complémentaires :

Acupuncture	Massage thérapeutique	Réflexologie
Aromathérapie (Huiles essentielles)	Médecine anthroposophique	Shiatsu
Art-thérapie	Médecine traditionnelle chinoise	Sophrologie
Homéopathie	Méditation	Tai chi
Hypnose	Ostéopathie	Yoga
Kinésiologie	Phytothérapie (thérapie par les plantes)	...

Traitement conventionnel : le traitement conventionnel englobe les thérapies telles que la chimiothérapie, la radiothérapie, la transplantation de cellules souches, la chirurgie et l'immunothérapie.

Remarque : dans ce questionnaire, le terme "médecines complémentaires" sera toujours utilisé au pluriel mais vos réponses peuvent très bien concerner une seule médecine complémentaire.

A1. Dans quelle tranche d'âge se trouve votre enfant actuellement?

- <2 ans ☐
- 2-4 ans ☐
- 5-7 ans ☐
- 8-12 ans ☐
- 13-18 ans ☐
- >18 ans ☐

A2. Depuis sa naissance, votre enfant a-t-il déjà utilisé des médecines complémentaires en lien ou Non avec sa maladie oncologique? Si oui, précisez laquelle/lesquelles dans la liste ci-dessous ? Si Non, cochez la première case "AUCUNE". Veuillez ne pas tenir compte des médecines complémentaires délivrées par un collaborateur du CHUV lors de votre séjour.

- AUCUNE ☐
- Acupuncture ☐
- Aromathérapie (huiles essentielles) ☐
- Art thérapie ☐
- Ayurveda ☐
- Biorésonance ☐
- Compléments alimentaires (vitamines, oligoéléments) ☐
- Fleurs de Bach ☐
- Homéopathie ☐
- Hypnose ☐
- Kinésiologie ☐
- Massage thérapeutique ☐
- Médecine anthroposophique (thérapie par le gui y compris) ☐
- Médecine traditionnelle chinoise (excepté acupuncture) ☐

Méditation ☐

Musicothérapie ☐

Ostéopathie ☐

Phytothérapie ☐

Réflexologie ☐

Sophrology ☐

Shiatsu ☐

Tai chi ☐

Yoga ☐

Autre ☐

A3. Veuillez écrire le-s nom-s des autres médecines complémentaires utilisées ci-dessous :

Médecine complémentaire 1	
Médecine complémentaire 2	
Médecine complémentaire 3	

Part B: Les médecines complémentaires et la maladie oncologique de votre enfant

B1. Votre enfant utilise-t-il des médecines complémentaires <u>en lien avec sa maladie oncologique</u>? Voici un rappel de quelques médecines complémentaires dans le tableau ci-dessous :

Acupuncture	Massage thérapeutique	Réflexologie
Aromathérapie (Huiles essentielles)	Médecine anthroposophique	Shiatsu
Art-thérapie	Médecine traditionnelle chinoise	Sophrologie
Homéopathie	Méditation	Tai chi
Hypnose	Ostéopathie	Yoga
Kinésiologie	Phytothérapie (thérapie par les plantes)	...

☐ Oui

☐ Non

☐ Je ne souhaite pas répondre

Part C: Les médecines complémentaires utilisées en fonction de la période de la maladie oncologique

C1. <u>Actuellement</u>, à quelle période de la maladie oncologique se trouve votre enfant ?

Si votre enfant se trouve en période de <u>rechute</u>, veuillez cocher la case "<u>Autre</u>"

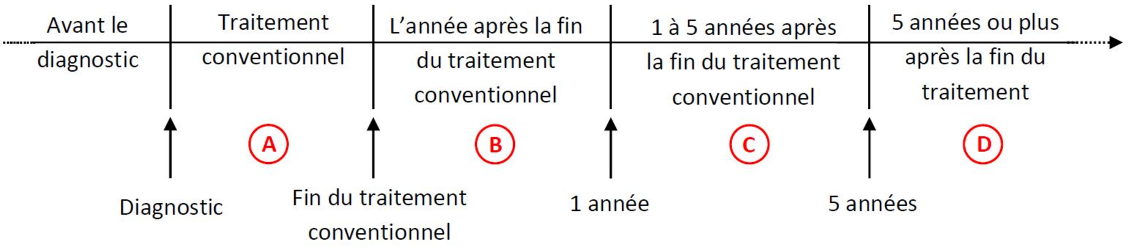

A ☐

B ☐

C ☐

D ☐

Autre ☐

C2. Si "Autre", veuillez préciser parmi ces deux propositions :

Information : le traitement de certains cancers de l'enfant comporte le risque de développer une autre maladie oncologique. N'hésitez pas à en parler à votre oncologue

Rechute ☐

Diagnostic d'une autre maladie oncologique ☐

C3. Pour chaque médecine complémentaire utilisée, veuillez indiquer <u>à</u> <u>quel-s moment-s</u> votre enfant y a eu recours.

	Avant le diagnostic	Durant le traitement oncologique	Durant l'année après la fin du traitement oncologique	Entre 1 et 5 années après la fin du traitement oncologique	≥5 années après la fin du traitement oncologique	Je ne me souviens pas	Je ne souhaite pas répondre
**Acupuncture**	☐	☐	☐	☐	☐	☐	☐
**Médecine anthroposophique**	☐	☐	☐	☐	☐	☐	☐
**Aromathérapie**	☐	☐	☐	☐	☐	☐	☐
**Art thérapie**	☐	☐	☐	☐	☐	☐	☐
**Ayurveda**	☐	☐	☐	☐	☐	☐	☐
**Biorésonance**	☐	☐	☐	☐	☐	☐	☐
**Fleurs de Bach**	☐	☐	☐	☐	☐	☐	☐
**Compléments alimentaires**	☐	☐	☐	☐	☐	☐	☐
**Phytothérapie**	☐	☐	☐	☐	☐	☐	☐
**Homéopathie**	☐	☐	☐	☐	☐	☐	☐
**Hypnose**	☐	☐	☐	☐	☐	☐	☐
**Kinésiologie**	☐	☐	☐	☐	☐	☐	☐
**Méditation**	☐	☐	☐	☐	☐	☐	☐
**Musicothérapie**	☐	☐	☐	☐	☐	☐	☐
**Ostéopathie**	☐	☐	☐	☐	☐	☐	☐
**Réflexologie**	☐	☐	☐	☐	☐	☐	☐
**Shiatsu**	☐	☐	☐	☐	☐	☐	☐
**Sophrology**	☐	☐	☐	☐	☐	☐	☐
**Tai chi**	☐	☐	☐	☐	☐	☐	☐
**Massage thérapeutique**	☐	☐	☐	☐	☐	☐	☐
**Médecine traditionnelle chinoise**	☐	☐	☐	☐	☐	☐	☐
**Yoga**	☐	☐	☐	☐	☐	☐	☐
**Autre médecine complémentaire 1**	☐	☐	☐	☐	☐	☐	☐
**Autre médecine complémentaire 2**	☐	☐	☐	☐	☐	☐	☐
**Autre médecine complémentaire 3**	☐	☐	☐	☐	☐	☐	☐

C3a. Pour chaque médecine complémentaire utilisée, veuillez indiquer <u>à quel-s moment-s</u> votre enfant y a eu recours, <u>avant la rechute</u>.

	Pas utilisée avant la rechute	Avant le diagnostic	Durant le traitement oncologique	Durant l'année après la fin du traitement oncologique	Entre 1 et 5 années après la fin du traitement oncologique	≥5 années après la fin du traitement oncologique	Je ne me souviens pas	Je ne souhaite pas répondre
**Acupuncture**	☐	☐	☐	☐	☐	☐	☐	☐
**Médecine anthroposophique**	☐	☐	☐	☐	☐	☐	☐	☐
**Aromathérapie**	☐	☐	☐	☐	☐	☐	☐	☐
**Art thérapie**	☐	☐	☐	☐	☐	☐	☐	☐
**Ayurveda**	☐	☐	☐	☐	☐	☐	☐	☐
**Biorésonance**	☐	☐	☐	☐	☐	☐	☐	☐
**Fleurs de Bach**	☐	☐	☐	☐	☐	☐	☐	☐
**Compléments alimentaires**	☐	☐	☐	☐	☐	☐	☐	☐
**Phytothérapie**	☐	☐	☐	☐	☐	☐	☐	☐
**Homéopathie**	☐	☐	☐	☐	☐	☐	☐	☐
**Hypnose**	☐	☐	☐	☐	☐	☐	☐	☐
**Kinésiologie**	☐	☐	☐	☐	☐	☐	☐	☐
**Méditation**	☐	☐	☐	☐	☐	☐	☐	☐
**Musicothérapie**	☐	☐	☐	☐	☐	☐	☐	☐
**Ostéopathie**	☐	☐	☐	☐	☐	☐	☐	☐
**Réflexologie**	☐	☐	☐	☐	☐	☐	☐	☐
**Shiatsu**	☐	☐	☐	☐	☐	☐	☐	☐
**Sophrology**	☐	☐	☐	☐	☐	☐	☐	☐
**Tai chi**	☐	☐	☐	☐	☐	☐	☐	☐
**Massage thérapeutique**	☐	☐	☐	☐	☐	☐	☐	☐
**Médecine traditionnelle chinoise**	☐	☐	☐	☐	☐	☐	☐	☐
**Yoga**	☐	☐	☐	☐	☐	☐	☐	☐
**Autre médecine complémentaire 1**	☐	☐	☐	☐	☐	☐	☐	☐
**Autre médecine complémentaire 2**	☐	☐	☐	☐	☐	☐	☐	☐
**Autre médecine complémentaire 3**	☐	☐	☐	☐	☐	☐	☐	☐

	C3b. Pour chaque médecine complémentaire utilisée, veuillez indiquer à quel-s moment-s votre enfant y a eu recours, après la rechute.							
	Pas utilisée après la rechute	Avant le diagnostic	Durant le traitement oncologique	Durant l'année après la fin du traitement oncologique	Entre 1 et 5 années après la fin du traitement oncologique	≥5 années après la fin du traitement oncologique	Je ne me souviens pas	Je ne souhaite pas répondre
**Acupuncture**	☐	☐	☐	☐	☐	☐	☐	☐
**Médecine anthroposophique**	☐	☐	☐	☐	☐	☐	☐	☐
**Aromathérapie**	☐	☐	☐	☐	☐	☐	☐	☐
**Art thérapie**	☐	☐	☐	☐	☐	☐	☐	☐
**Ayurveda**	☐	☐	☐	☐	☐	☐	☐	☐
**Biorésonance**	☐	☐	☐	☐	☐	☐	☐	☐
**Fleurs de Bach**	☐	☐	☐	☐	☐	☐	☐	☐
**Compléments alimentaires**	☐	☐	☐	☐	☐	☐	☐	☐
**Phytothérapie**	☐	☐	☐	☐	☐	☐	☐	☐
**Homéopathie**	☐	☐	☐	☐	☐	☐	☐	☐
**Hypnose**	☐	☐	☐	☐	☐	☐	☐	☐
**Kinésiologie**	☐	☐	☐	☐	☐	☐	☐	☐
**Méditation**	☐	☐	☐	☐	☐	☐	☐	☐
**Musicothérapie**	☐	☐	☐	☐	☐	☐	☐	☐
**Ostéopathie**	☐	☐	☐	☐	☐	☐	☐	☐
**Réflexologie**	☐	☐	☐	☐	☐	☐	☐	☐
**Shiatsu**	☐	☐	☐	☐	☐	☐	☐	☐
**Sophrology**	☐	☐	☐	☐	☐	☐	☐	☐
**Tai chi**	☐	☐	☐	☐	☐	☐	☐	☐
**Massage thérapeutique**	☐	☐	☐	☐	☐	☐	☐	☐
**Médecine traditionnelle chinoise**	☐	☐	☐	☐	☐	☐	☐	☐
**Yoga**	☐	☐	☐	☐	☐	☐	☐	☐
**Autre médecine complémentaire 1**	☐	☐	☐	☐	☐	☐	☐	☐
**Autre médecine complémentaire 2**	☐	☐	☐	☐	☐	☐	☐	☐
**Autre médecine complémentaire 3**	☐	☐	☐	☐	☐	☐	☐	☐

	C3c. Pour chaque médecine complémentaire utilisée, veuillez indiquer à quel-s moment-s votre enfant y a eu recours, avant la rechute <u>avant le diagnostic d'une autre maladie oncologique</u>							
	Pas utilisée avant le diagnostic d'une autre maladie oncologique	Avant le diagnostic	Durant le traitement oncologique	Durant l'année après la fin du traitement oncologique	Entre 1 et 5 années après la fin du traitement oncologique	≥5 années après la fin du traitement oncologique	Je ne me souviens pas	Je ne souhaite pas répondre
**Acupuncture**	☐	☐	☐	☐	☐	☐	☐	☐
**Médecine anthroposophique**	☐	☐	☐	☐	☐	☐	☐	☐
**Aromathérapie**	☐	☐	☐	☐	☐	☐	☐	☐
**Art thérapie**	☐	☐	☐	☐	☐	☐	☐	☐
**Ayurveda**	☐	☐	☐	☐	☐	☐	☐	☐
**Biorésonance**	☐	☐	☐	☐	☐	☐	☐	☐
**Fleurs de Bach**	☐	☐	☐	☐	☐	☐	☐	☐
**Compléments alimentaires**	☐	☐	☐	☐	☐	☐	☐	☐
**Phytothérapie**	☐	☐	☐	☐	☐	☐	☐	☐
**Homéopathie**	☐	☐	☐	☐	☐	☐	☐	☐
**Hypnose**	☐	☐	☐	☐	☐	☐	☐	☐
**Kinésiologie**	☐	☐	☐	☐	☐	☐	☐	☐
**Méditation**	☐	☐	☐	☐	☐	☐	☐	☐
**Musicothérapie**	☐	☐	☐	☐	☐	☐	☐	☐
**Ostéopathie**	☐	☐	☐	☐	☐	☐	☐	☐
**Réflexologie**	☐	☐	☐	☐	☐	☐	☐	☐
**Shiatsu**	☐	☐	☐	☐	☐	☐	☐	☐
**Sophrology**	☐	☐	☐	☐	☐	☐	☐	☐
**Tai chi**	☐	☐	☐	☐	☐	☐	☐	☐
**Massage thérapeutique**	☐	☐	☐	☐	☐	☐	☐	☐
**Médecine traditionnelle chinoise**	☐	☐	☐	☐	☐	☐	☐	☐
**Yoga**	☐	☐	☐	☐	☐	☐	☐	☐
**Autre médecine complémentaire 1**	☐	☐	☐	☐	☐	☐	☐	☐
**Autre médecine complémentaire 2**	☐	☐	☐	☐	☐	☐	☐	☐
**Autre médecine complémentaire 3**	☐	☐	☐	☐	☐	☐	☐	☐

	C3d. Pour chaque médecine complémentaire utilisée, veuillez indiquer à quel-s moment-s votre enfant y a eu recours, avant la rechute <u>après le diagnostic d'une autre maladie oncologique</u>							
	Pas utilisée après le diagnostic d'une autre maladie oncologique	Avant le diagnostic	Durant le traitement oncologique	Durant l'année après la fin du traitement oncologique	Entre 1 et 5 années après la fin du traitement oncologique	≥5 années après la fin du traitement oncologique	Je ne me souviens pas	Je ne souhaite pas répondre
**Acupuncture**	☐	☐	☐	☐	☐	☐	☐	☐
**Médecine anthroposophique**	☐	☐	☐	☐	☐	☐	☐	☐
**Aromathérapie**	☐	☐	☐	☐	☐	☐	☐	☐
**Art thérapie**	☐	☐	☐	☐	☐	☐	☐	☐
**Ayurveda**	☐	☐	☐	☐	☐	☐	☐	☐
**Biorésonance**	☐	☐	☐	☐	☐	☐	☐	☐
**Fleurs de Bach**	☐	☐	☐	☐	☐	☐	☐	☐
**Compléments alimentaires**	☐	☐	☐	☐	☐	☐	☐	☐
**Phytothérapie**	☐	☐	☐	☐	☐	☐	☐	☐
**Homéopathie**	☐	☐	☐	☐	☐	☐	☐	☐
**Hypnose**	☐	☐	☐	☐	☐	☐	☐	☐
**Kinésiologie**	☐	☐	☐	☐	☐	☐	☐	☐
**Méditation**	☐	☐	☐	☐	☐	☐	☐	☐
**Musicothérapie**	☐	☐	☐	☐	☐	☐	☐	☐
**Ostéopathie**	☐	☐	☐	☐	☐	☐	☐	☐
**Réflexologie**	☐	☐	☐	☐	☐	☐	☐	☐
**Shiatsu**	☐	☐	☐	☐	☐	☐	☐	☐
**Sophrology**	☐	☐	☐	☐	☐	☐	☐	☐
**Tai chi**	☐	☐	☐	☐	☐	☐	☐	☐
**Massage thérapeutique**	☐	☐	☐	☐	☐	☐	☐	☐
**Médecine traditionnelle chinoise**	☐	☐	☐	☐	☐	☐	☐	☐
**Yoga**	☐	☐	☐	☐	☐	☐	☐	☐
**Autre médecine complémentaire 1**	☐	☐	☐	☐	☐	☐	☐	☐
**Autre médecine complémentaire 2**	☐	☐	☐	☐	☐	☐	☐	☐
**Autre médecine complémentaire 3**	☐	☐	☐	☐	☐	☐	☐	☐

C4. <u>Actuellement</u>, quel-s traitement-s oncologique-s conventionnel-s votre enfant suit-il ?

Chimiothérapie ☐

Immunothérapie ☐

Radiothérapie ☐

Chirurgie ☐

Transplantation de cellules souches ☐

Autre ☐

Autre

Part D: Les médecines complémentaires et l'oncologue en charge de votre enfant

D1. Lors d'une consultation, avez-vous eu <u>une discussion au sujet des médecines complémentaires</u> avec l'oncologue?

Oui ☐

Non ☐

Je ne me souviens pas ☐

Je ne souhaite pas répondre ☐

D2. Qui a commencé cette discussion au sujet des médecines complémentaires ?

Moi (La mère et/ou le père) ☐

L'oncologue pédiatrique ☐

Mon enfant ☐

Autre ☐

Autre

[ ]

D3. L'oncologue est-il/elle au courant que vous utilisez des médecines complémentaires pour votre enfant <u>dans le cadre de sa maladie</u> <u>oncologique</u> ?

Oui ☐

Non ☐

Je ne me souviens pas ☐

Je ne souhaite pas répondre ☐

D4. Comment <u>l'oncologue</u> a-t-il/elle réagi lorsque vous lui avez dit que vous utilisez des médecines complémentaires pour votre enfant ?

Il/elle m'a encouragé-e à continuer le traitement de médecines complémentaires. ☐

Il/elle m'a demandé d'arrêter le traitement de médecines complémentaires. ☐

Il/elle m'a procuré des informations et expliqué la/les contre-indication-s. ☐

Il/elle n'a pas fait de recommandation. ☐

Il/elle n'a pas souhaité en parler. ☐

Autre ☐

Autre

D5. Pourquoi l'oncologue n'a pas été mis-e au courant que vous utilisez des médecines complémentaires pour votre enfant ?

Parce qu'il/elle ne me l'a pas demandé. ☐

Parce qu'il/elle ne comprendrait pas. ☐

Parce qu'il/elle ne serait pas content-e. ☐

Parce que j'ai oublié de le mentionner. ☐

Parce que cela n'a pas d'importance de le lui dire. ☐

Autre ☐

Autre

D6. L'oncologue vous <u>a-t-il/elle proposé</u> d'utiliser des médecines complémentaires pour votre enfant <u>dans le cadre de sa maladie oncologique</u>?

Oui ☐

Non ☐

Je ne me souviens pas ☐

Je ne souhaite pas répondre ☐

D7. Souhaiteriez-vous avoir accès à <u>une consultation spécialisée de conseils et de soins en médecines complémentaires</u> au CHUV pour votre enfant <u>dans le cadre de sa maladie oncologique</u> ?

Oui ☐

Non ☐

Je ne sais pas ☐

Je ne souhaite pas répondre ☐

D8. Pour chacune des phrases ci-dessous (a-e), indiquez la réponse représentant le mieux <u>votre opinion</u>.

	Pas du tout d'accord	Pas d'accord	Ni d'accord ni pas d'accord	D'accord	Tout à fait d'accord	Je ne me souviens pas	Je ne souhaite pas répondre
**a.** Je serais prêt-e à essayer une méthode de médecine complémentaire proposée par l'oncologue en complément du traitement conventionnel pour mon enfant.	☐	☐	☐	☐	☐	☐	☐
**b.** J'ai reçu suffisamment d'informations de l'oncologue sur les bénéfices des médecines complémentaires.	☐	☐	☐	☐	☐	☐	☐
**c.** J'ai reçu suffisamment d'informations de l'oncologue sur les risques des médecines complémentaires.	☐	☐	☐	☐	☐	☐	☐
**d.** Recourir à des médecines complémentaires peut interagir avec le traitement conventionnel de mon enfant.	☐	☐	☐	☐	☐	☐	☐
**e.** Recourir à des médicaments et/ou traitements à base de plantes peut interagir avec le traitement conventionnel de mon enfant.	☐	☐	☐	☐	☐	☐	☐

Part E: Les sources d'information utilisées pour se renseigner sur les médecines complémentaires

E1. Dans la liste ci-dessous, indiquez <u>le-s moyen-s</u> qui vous a/ont permis de vous renseigner sur les médecines complémentaires ? Précisez si vous l'/les avez <u>"peu"</u> ou <u>"souvent"</u> consulté-s.

**Thérapeute complémentaire (non médecin) comprend : homéopathe non médecin, ostéopathe, naturopathe, ...*

	Pas consulté	Peu consulté	Souvent consulté	Je ne souhaite pas répondre
Ami-e ou famille	☐	☐	☐	☐
Association de parents	☐	☐	☐	☐
Infirmier-ère spécialiste en oncologie pédiatrique	☐	☐	☐	☐
Médecin spécialiste en oncologie pédiatrique	☐	☐	☐	☐
Médecin (de famille, généraliste, ...)	☐	☐	☐	☐
Médecin formé en médecines complémentaires (homéopathe, acupuncteur,	☐	☐	☐	☐
Média (internet, journal, livre, télévision, radio, publicité)	☐	☐	☐	☐
Pharmacien-ne	☐	☐	☐	☐
Thérapeute complémentaire (non médecin)*	☐	☐	☐	☐

Part F: Les informations générales

F1. Qui a rempli ce questionnaire ?

- La mère ☐
- Le père ☐
- L'enfant ☐
- Un autre membre de la famille ☐
- Autre ☐

F2. Votre enfant dispose-t-il d'une assurance complémentaire pour les médecines complémentaires ?

- Oui ☐
- Non ☐
- Je ne sais pas ☐
- Je ne souhaite pas répondre ☐

F3. Pour quelle-s raison-s votre enfant ne dispose-t-il pas d'une assurance complémentaire pour les médecines complémentaires ?

- L'assurance complémentaire pour les médecines complémentaires a été refusée par l'assurance. ☐
- Je ne dispose pas des ressources financières nécessaires. ☐
- Je ne connais pas ce type d'assurance complémentaire. ☐
- Je ne souhaite pas souscrire une telle assurance pour mon enfant. ☐
- Je ne sais pas. ☐
- Autre ☐

Autre

Part G: Pour terminer ...

Les deux dernières questions ci-dessous vous permettent de vous exprimer sur les médecines complémentaires en général et sur le questionnaire.

Pour terminer le questionnaire, merci de cliquer sur le bouton "Envoyer" en bas de la page.

G1. Avez-vous des commentaires sur la thématique des médecines complémentaires ?

G2. Avez-vous des commentaires sur ce questionnaire ?

G3. Souhaiteriez-vous être informé-e des résultats de notre étude ?

Les résultats de cette étude seront disponibles dans quelques mois, une fois l'analyse des données terminée. Ils feront également l'objet d'une publication dans une revue scientifique.

Oui ☐

Non ☐

G4. Merci d'inscrire votre adresse e-mail ci-dessous afin que nous puissions vous contacter, si vous souhaitez être informé-e des résultats.

Votre adresse e-mail sera utilisée uniquement pour vous envoyer les résultats de notre étude. La transmission de votre adresse e-mail ne remet en aucun cas en cause la confidentialité de vos réponses.
